# Local and Systemic Cardiovascular Effects from Monochromatic Infrared Therapy in Patients with Knee Osteoarthritis: A Double-Blind, Randomized, Placebo-Controlled Study

**DOI:** 10.1155/2012/583016

**Published:** 2012-06-27

**Authors:** Ru-Lan Hsieh, Wei-Cheng Liao, Wen-Chung Lee

**Affiliations:** ^1^Department of Physical Medicine and Rehabilitation, Shin Kong Wu Ho-Su Memorial Hospital, 95 Wen-Chang Road, Shih-Lin District, Taipei 111, Taiwan; ^2^School of Medicine, College of Medicine, Taipei Medical University, 250 Wuxing Street, Taipei 110, Taiwan; ^3^School of Nursing and Management in Gerontology, College of Nursing, Taipei Medical University, 250 Wuxing Street, Taipei 110, Taiwan; ^4^Institute of Epidemiology and Preventive Medicine, College of Public Health, National Taiwan University, 17 Xuzhou Road, Taipei 100, Taiwan

## Abstract

Infrared (IR) therapy is used for pain relief in patients with knee osteoarthritis (OA). However, IR's effects on the cardiovascular system remain uncertain. Therefore, we investigated the local and systemic cardiovascular effects of monochromatic IR therapy on patients with knee OA in a double-blind, randomized, placebo-controlled study. Seventy-one subjects with knee OA received one session of 40 min of active or placebo monochromatic IR treatment (with power output of 6.24 W, wavelength of 890 nm, power density of 34.7 mW/cm^2^ for 40 min, total energy of 41.6 J/cm^2^ per knee per session) over the knee joints. Heart rate, blood pressure, and knee arterial blood flow velocity were periodically assessed at the baseline, during, and after treatment. Data were analyzed by repeated-measure analysis of covariance. Compared to baseline, there were no statistically significant group x time interaction effects between the 2 groups for heart rate (*P* = 0.160), blood pressure (systolic blood pressure: *P* = 0.861; diastolic blood pressure: *P* = 0.757), or mean arterial blood flow velocity (*P* = 0.769) in follow-up assessments. The present study revealed that although there was no increase of knee arterial blood flow velocity, monochromatic IR therapy produced no detrimental systemic cardiovascular effects.

## 1. Introduction

Osteoarthritis (OA) generally involves articular cartilage, anabolic and catabolic mechanisms, and bony structures in the synovial joints [1]. Weaker quadriceps muscle strength, lower knee proprioception, and poor balance with increased postural swaying were noted in subjects with knee OA than in age- and gender-matched controls [2]. Pain and decreased postural stability may be accompanied by difficulties in performing basic and instrumental daily activities, increased fall risks among community-dwelling elderly [3, 4], and a decreased quality of life [5].

Physical modalities are commonly used to treat older patients with knee OA to ameliorate pain and improve functional performance in the rehabilitation medical field. Physical modalities, such as hot packs, pulse ultrasound, transcutaneous electrical nerve stimulation, and phototherapy, are commonly applied to patients with musculoskeletal pain to increase local circulation [6]. However, there are few high-quality clinical studies with randomized placebo-controlled designs on physical modalities' therapeutic effects in the rehabilitation medicine field [7].

Light encompasses a portion of the electromagnetic spectrum. Infrared (IR) radiation wavelengths range from 750 nm to 1 mm. In 2002, the US Food and Drug Administration approved IR therapy for pain relief associated with neck and head pain, arthritis, and carpal tunnel syndrome [8]. IR therapy is commonly used for patients with wounds, lower-limb peripheral neuropathies, and musculoskeletal disorders such as knee OA [7, 9–19]. Photoenergy exerts bioenergetic, biostimulating, biochemical, and bioelectrical effects on cells [20, 21]. The biological effect of phototherapy is related to photochemical cellular reactions rather than thermal reactions [22]. Phototherapy has been found to improve microcirculation by increasing arterioles diameter and blood flow velocity [23–25]. Improving microcirculation at the local and systemic levels is one of the most important phototherapy effects [26, 27]. It is speculated that vessel dilatation, increase of blood flow rate, and improved blood rheologic properties are mediated by NO, prostacyclin, and endothelial-derived hyperpolarizing factors, all of which are produced by endothelial cells [26, 28]. NO causes rapid transduction and increases local blood flow followed by prostacyclin and endothelial-derived hyperpolarizing factors in changing microcirculation at the systemic level [26, 29]. In addition to mediation by enhancing NO synthesis and increasing microcirculation, phototherapy also relives pain by other pathways and mechanisms, such as by modulating inhibitory cyclooxygenase and prostaglandin E2, modulating nerve transmission, increasing endorphin and serotonin release, and stimulating metabolism [8, 21, 30].

A series of IR treatments had been confirmed to have significant efficacy in improving pain, function, and quality of life in patients with knee OA [17, 31, 32]. Possible mechanisms include peripheral nerve stimulation, microcirculation enhancement, analgesic effects, inflammation resolution, chondrocyte proliferation enhancement, and increased matrix synthesis [17, 33]. Due to significant efficacy of OA knee treatment with IR therapy as a series of sessions, it is necessary to provide evidence that IR therapy does not produce any detrimental systemic cardiovascular effect. However, to our knowledge, no comprehensive study has focused on IR therapy's cardiovascular effects in patients with knee OA [32, 34, 35].

If IR therapy in patients with knee OA can improve the knee arterial blood flow velocity without producing detrimental systemic cardiovascular effects, then the increased blood flow in and/or around the knee joint may infer benefits to the knee joint such as pain reduction in patients with knee OA by long term, repeated IR treatments. Therefore, we hypothesized that IR therapy presumably would influence knee joint tissue perfusion by increasing the local arterial blood velocity at the knee without producing detrimental systemic cardiovascular effects. In our research, we conducted a double-blind, randomized, and placebo-controlled study to examine local and systemic cardiovascular effects from monochromatic IR therapy in patients with knee OA.

## 2. Materials and Methods

This study was conducted at Shin Kong Wu Ho-Su Memorial Hospital, a teaching hospital with 921 beds located in northern Taiwan. In total, 73 subjects confirmed to have knee OA were identified and recruited from the clinic of the Department of Physical Medicine and Rehabilitation at the hospital. All patients fulfilled the combined knee OA clinical and radiographic criteria established by the American College of Rheumatology [36]. Anteroposterior radiographic views of both knees were taken while bearing weight. A qualified senior physiatrist was in charge of reading the X-rays to classify subjects' Kellgren-Lawrence scores. The hospital's Institutional Review Board for the Protection of Human Subjects approved this study. Written informed consent was obtained from each subject. Subjects with a history of stroke, peripheral vascular disease, peripheral neuropathy, a previous knee operation with an implant, a malignancy, or who were pregnant or planning to become pregnant were excluded.

General information, including age, gender, educational level, marital status, work status, smoking and drinking habits, and comorbidities, was recorded. The body mass index (BMI) was calculated. The self-reported OA knee-specific health status was assessed with the Chinese version of the Western Ontario and McMaster University Osteoarthritis Index (WOMAC) [37]. Using a visual analog scale, total WOMAC scores of pain, stiffness, and physical function, respectively vary from 0 to 500, 0 to 200, and 0 to 1700. Higher scores represent worse symptoms with greater functional limitation. The reliability and validity of the three visual analog scale versions are excellent [37–40].

**Figure 1:**
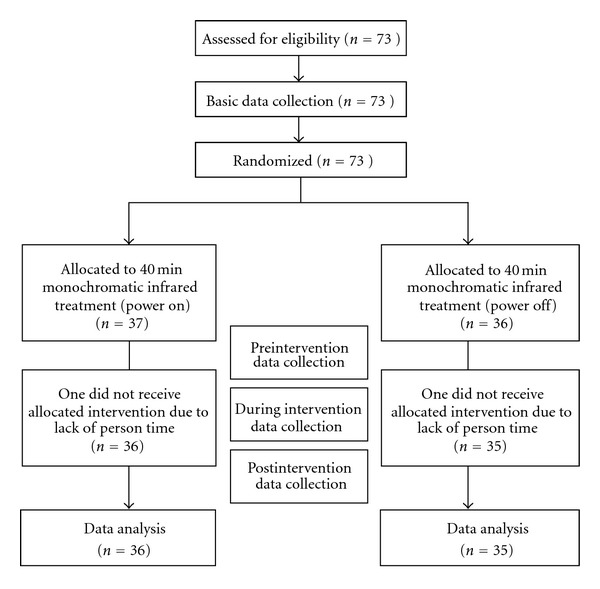
Consort flow diagram.

After completing basic data recording, patients were allocated to a treatment group (active treatment) or a placebo group (inactive treatment) following the block randomization principle (with a block size of four). The allocation was initially concealed. An envelope was opened for each consecutive subject to reveal his or her group assignment at the time when he or she was recruited to the study. All patients, regardless of group assignment, underwent 40 min of monochromatic IR therapy with either power on (treatment group) or power off (placebo group) (Figure 1).

Each subject laid down on a standard bed with socks, shoes, and pants removed and rested for 15 min before the intervention in a quiet room with air conditioning. An Anodyne Therapy Professional System (Anodyne Therapy Professional System 480) was used in this study. The device has a main power unit with 8 flexible therapy pads. Each pad contains 60 superluminous gallium-aluminum arsenide diodes that emit an 890 nm light energy wavelength. Eight therapeutic pads were used in this study for both knees, and subjects in the treatment group received a total energy of 41.6 J/cm^2^ per knee per session (with a radiant power output of 6.24 W, at a wavelength of 890 nm, and a power density of 34.7 mW/cm^2^ for 40 min).

**Figure 2:**
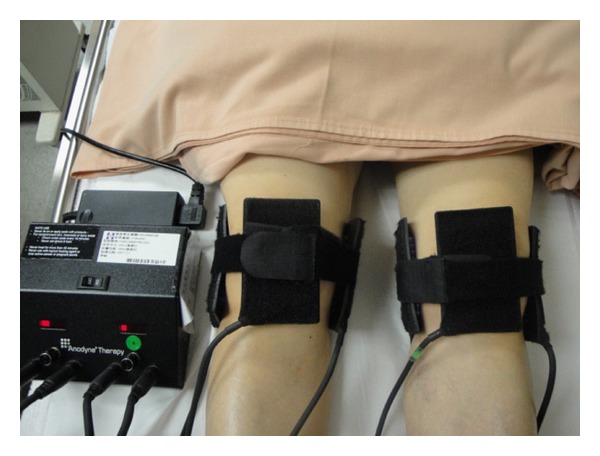
Monochromatic infrared therapy application.

Four therapy pads were placed over the following sites in each knee: the anterior knee joint, the posterior knee joint, and the medial and lateral knee joints (Figure 2). The pads were held in place with neoprene straps supplied by the manufacturer. All subjects were told that they may or may not feel anything from the treatment. Subjects received 1 session of monochromatic IR therapy for 40 min with either the power on or off. The manufacturer checked the monochromatic IR device before the intervention began on the first participant.

The heart rate and blood pressure were measured over the brachial artery with an automated sphygmomanometer (Tango^+^ Stress BP, Sun Tech Medical Instruments) by a well-trained technician. The blood pressure measurement had good reliability and validity [41–43]. The instrument was calibrated, and the same cuff was used for all subjects. The heart rate was monitored before treatment, immediately after completing 40 min of treatment, and 5, 10, and 15 min after completing treatment. Systolic and diastolic blood pressures were automatically recorded before monochromatic IR treatment, every 10 min during the 40 min of treatment, and 5 and 15 min after completing treatment. No conversation was allowed between the participants and the technician during the whole course of heart rate and blood pressure measurements.

Color Doppler ultrasonography (LOGIQ P5, GE Ultrasound Korea, General Electric) was performed on patients in a prone position by a qualified senior physiatrist who was not informed of each patient's group allocation. The peak popliteal arterial systolic blood flow velocity (meters/second) was measured in each subject by high-resolution B-mode ultrasound images using standardized parameters with a 7.5 MHz linear array transducer. The peak popliteal arterial blood flow velocity was measured before IR radiation treatment; immediately after completion of 40 min of treatment; and 5, 10, and 15 minutes after treatment. It has high reliability [44, 45].

Except for the physical therapist performing the monochromatic IR treatments, neither the subjects receiving the treatment nor the investigators (including the technician who measured patients' heart rates and blood pressures, and the physiatrist who conducted the Doppler study) were aware of the monochromatic IR therapy's operating status during the study's treatment and data collection periods.

The results are expressed as the mean ± the standard deviation. A chi-squared test or *t*-test was used to analyze demographic data such as age, gender, educational level, marital status, occupation, comorbidities, smoking and drinking habits, BMI, Kellgren-Lawrence scores, and knee OA-specific measures of pain, stiffness, and physical function of the treatment and placebo groups. Repeated-measure analysis of covariance (ANCOVA) was used to assess the heart rate, systolic and diastolic blood pressures, and mean arterial knee joint blood flow in patients with knee OA between the follow-up assessments in each group, using the pretreatment baseline as the covariate. The group effect, time effect, and group x time interaction effect for the 2 groups at the follow-up assessments were analyzed. We report the results of the ANCOVA by providing the *F* statistic, degrees of freedom, and the *P* value for all 71 participants. The level of statistical significance was set at *P* < 0.05.

## 3. Results

Seventy-three subjects were enrolled in this study. Two subjects refused to participate after completing basic data collection due to personal time constraints. There was no statistically significant difference in the 2 groups in age, gender, educational level, marital status, occupation, comorbidities, smoking and drinking habits, BMI, or severity of knee OA according to the Kellgren-Lawrence scores and WOMAC assessments. Detailed demographic data for both groups are shown in Table 1.

**Figure 3:**
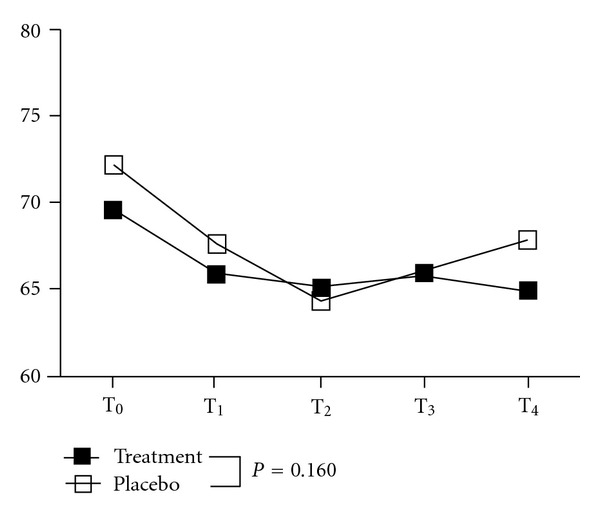
Changes in the heart rate with monochromatic infrared treatment. Solid square: treatment group; hollow square: placebo group; T_0_: before treatment; T_1_: after 40 min of treatment; T_2_: 5 min after treatment; T_3_: 10 min after treatment; T_4_: 15 min after treatment. Between groups by repeated-measure ANCOVA: group effect: *P* = 0.918 (*F*_1,68_ = 0.01); time effect: *P* = 0.340 (*F*_3,204_ = 1.12); group x time interaction effect: *P* = 0.160 (*F*_3,204_ = 1.74).

Compared to pretreatment, there was no statistical significance demonstrated in the heart rate between the 2 groups (group effect: *P* = 0.918; time effect: *P* = 0.340; group x time interaction effect: *P* = 0.160) during the 4 follow-up assessments (after 40 min of treatment; and 5, 10, and 15 min after treatment) (Table 2, Figure 3).

**Figure 4:**
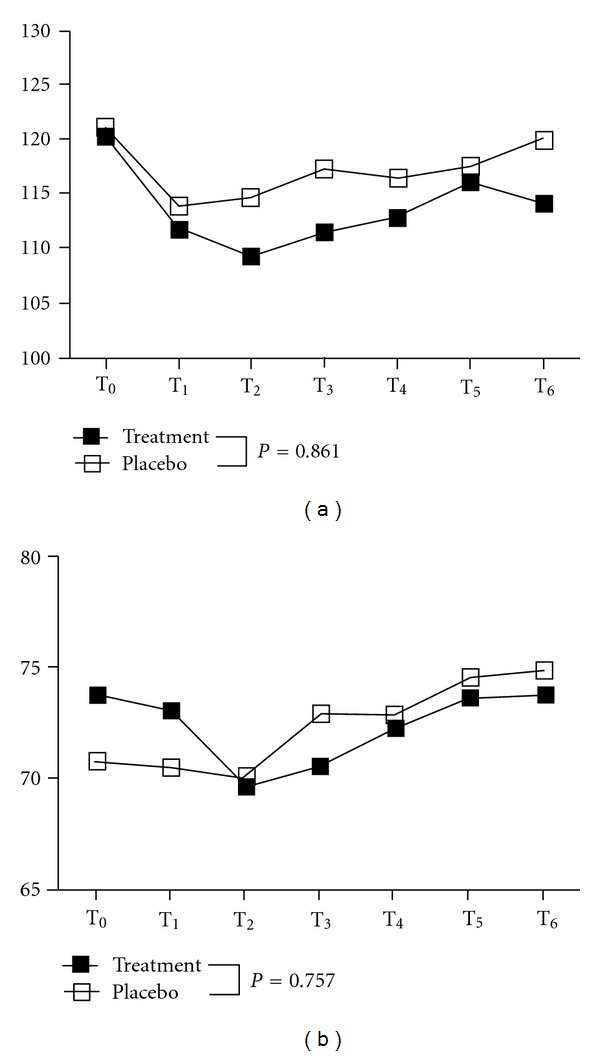
Changes in blood pressure with monochromatic infrared treatment. Solid square: treatment group; hollow square: placebo group; T_0_: before treatment; T_1_: after 10 min of treatment; T_2_: after 20 min of treatment; T_3_: after 30 min of treatment; T_4_: after 40 min of treatment; T_5_: 5 min after treatment; T_6_: 15 min after treatment. (a) systolic blood pressure; (b) diastolic blood pressure. Between groups by repeated-measure ANCOVA: systolic blood pressure: group effect: *P* = 0.281 (*F*_1,68_ = 1.18); time effect: *P* = 0.180 (*F*_5,335_ = 1.53); group x time interaction effect: *P* = 0.861 (*F*_5,335_ = 0.38); diastolic blood pressure: group effect: *P* = 0.262 (*F*_1,68_ = 0.19); time effect: *P* = 0.663 (*F*_5,335_ = 0.65); group x time interaction effect: *P* = 0.757 (*F*_5,335_ = 0.53).

 As shown in Table 2 and Figure 4, there was no statistically significant difference in systolic blood pressure (group effect: *P* = 0.281; time effect: *P* = 0.180; group x time interaction effect: *P* = 0.861) or diastolic blood pressure (group effect: *P* = 0.262; time effect: *P* = 0.663; or group x time interaction effect: *P* = 0.757) between the 2 groups during the 6 follow-up assessments (after 10, 20, 30, and 40 min of the treatment; and 5 and 10 min after the treatment was completed).

**Figure 5:**
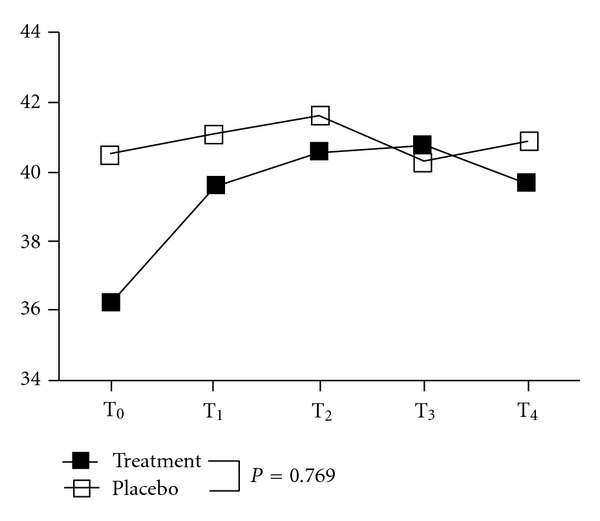
Changes in the blood flow velocity with monochromatic radiation treatment. Solid square: treatment group; hollow square: placebo group; T_0_: before treatment; T_1_: after 40 min of treatment; T_2_: 5 min after treatment; T_3_: 10 min after treatment; T_4_: 15 min after treatment. Between groups by repeated-measure ANCOVA: group effect: *P* = 0.666 (*F*_1,68_ = 0.19); time effect: *P* = 0.323 (*F*_3,204_ = 1.17); group x time interaction effect: *P* = 0.769 (*F*_3,204_ = 0.38).

 As for the popliteal arterial blood flow velocity, compared to pretreatment, there was no statistically significant difference in the blood flow (group effect: *P* = 0.666, time effect: *P* = 0.323, group x time interaction effect: *P* = 0.769) at the 4 separate follow-up assessments between the 2 groups (after 40 min of treatment; and 5, 10, and 15 min after treatment) (Table 2, Figure 5).

No local or systemic side effects were reported during or after the intervention.

## 4. Discussion

A series of IR treatments are demonstrated to have significant efficacy in improving pain in patients with knee OA. However, the cardiovascular effects by these treatments remain uncertain. To our knowledge, this is the first study to demonstrate the local and systemic cardiovascular effects of monochromatic IR therapy in patients with knee OA. Our results revealed that although there was no knee arterial blood flow velocity increase, monochromatic IR therapy produced no detrimental systemic cardiovascular effects.

A significant microcirculation increase began after 20 min of IR therapy and reached a maximal level 15 min after treatment termination [46]. Therefore, we conducted 40 min of monochromatic IR therapy and followed up for 15 min after treatment termination to examine the local and systemic cardiovascular effects in patients with knee OA in the present study. There has been a tendency to shift from treatment with laser-based devices to treatment by light-emitting diodes in recent years due to the lower cost, lack of coherence, and larger spot size in light-emitting diode devices [8, 47, 48]. Therefore, we used the light-emitting diodes in this study. Color Doppler sonography was used for local blood flow velocity evaluation, and it is widely used in clinical medicine because it is a rapid, simple, accurate, and noninvasive method of objectively monitoring blood flow [49, 50]. However, this study did not demonstrate an increase in the local arterial blood flow velocity after 40 min of monochromatic IR therapy over the knee joints in patients with knee OA.

Measuring blood pressure with a conventional manual sphygmomanometer used by a physician in routine clinical practice often reported inconsistent and imprecise blood pressure readings due to patient-physician interaction, failure to minimize patient anxiety, or poor measurement techniques [51]. The “white coat” bias has been demonstrated to be 15% to 20% in patients with hypertension [52]. Therefore, blood pressure measurement taken outside the clinic using ambulatory blood pressure monitoring is the gold standard measure [53]. However, due to cost and convenience considerations, automatic devices are commonly used in clinics for blood pressure measurements.

Measuring blood pressure using an automatic device in a clinic and leaving the patient alone in a quiet room for at least 14 minutes rest before measurement were found to minimize the white coat effect and yielded values that were comparable to the ambulatory blood pressure measurements [54–56]. The most innovative features to measure blood pressure by automatic devices were that the cuff must be wrapped snugly around the arm, and the patient must keep proper posture during measurement [57]. Therefore, in this study, to avoid the resting effect on heart rate and blood pressure (as the subjects were lying down for up to 60 min for treatment and followup) and the white coat effect, all participants were asked to lie on the bed in a quiet room for 15 min before beginning the blood pressure measurement and intervention. The blood pressure measuring point was 8 cm above the right elbow joint [57], and all measurements were taken on the right arm. A technician rather than a physician completed the blood pressure measurement. In terms of heart rate and blood pressure, there was no significant difference between the treatment group and placebo group during the 40 min of treatment or 10 to 15 min after treatment termination.

Compared to a previously conducted, community-based cohort study in Taiwan [58], although the mean age of participants was relatively older, the systolic blood pressure and the diastolic blood pressure mean value ranges were relatively lower in the present study (the respective values for the previously-conducted study in contrast to the present study are as follows: mean age (years): 56.3 in contrast to 61.2; mean heart rate (beats/min): 64.8 in contrast to 67.1; mean systolic blood pressure (mmHg): 120.2 in contrast to 115.4; mean diastolic blood pressure (mmHg): 74.1 in contrast to 72.4). In contrast to participants' blood pressures being measured by a conventionl, manual sphygmomanometer in a clinic by a physician without mentioning full rest as in the previous study, the participants' blood pressure monitors were applied by a technician, blood pressure was measured automatically in a quiet room after resting for 15 minutes, and conversation between the technician and participants during heart rate and blood pressure measurements was prohibited in the current study. All of these procedures would effectively minimize the white coat effect and measure blood pressure more accurately. This could partially explain why the ranges of systolic and diastolic blood pressure are lower than the average ranges for 56 years old.

Although all participants were told that they may or may not feel anything from monochromatic IR therapy, active monochromatic IR therapy actually emits mild, tangible heat. Therefore, subjects would expect a difference in perception from monochromatic IR therapy between the 2 groups, which could have given subjects in the treatment group a greater perception that they were being treated in contrast to those in the control group. The percentage of patients who perceived heat in the control group in contrast to the experimental group was 33% versus 58% (*P* = 0.101). Stratified analysis according to whether the participants perceived heat feeling or not was further performed to control for this factor. For the participants who perceived a heat feeling, we found statistically significant group x time interactions for heart rate, with higher trends in the control group (*P* = 0.033), and no statistically significant group effect (*P* = 0.762) or time effect (*P* = 0.708). There was no statistically significant group x time interaction effect for systolic blood pressure (*P* = 0.543), diastolic blood pressure (*P* = 0.940), or knee arterial blood flow velocity (*P* = 0.323). For participants who did not perceive a heat feeling, there was no significant group x time interaction for heart rate (*P* = 0.523), systolic blood pressure (*P* = 0.779), diastolic blood pressure (*P* = 0.574), or knee arterial blood flow velocity (*P* = 0.444). Although the perceived heat feeling by active monochromatic IR therapy could compromise the experiment's blindness on the patients' side, it does not affect the results after we had controlled for that factor.

Because the effects of monochromatic IR therapy are time-dependent [59], the level of photoenergy delivered would have affected the study's results. Compared to previous studies that applied total energies of 52.0–58.5 J/cm^2^ [11, 18, 19], the present study used 34.7 mW/cm^2^ for 40 min for a higher total energy of 83.2 J/cm^2^. At this higher energy, monochromatic IR therapy still had no detrimental systemic cardiovascular effects on patients with knee OA as measured by the heart rate and blood pressure.

OA is often associated with obesity and several cardiovascular conditions, including coronary artery disease, hypertension, and diabetes mellitus [60]. Because obesity, hypertension, and cardiovascular diseases are present in metabolic syndrome, it is hypothesized that OA may represent another aspect of metabolic syndrome [61, 62]. Potential mechanisms for joint OA include the following: (1) reduced blood flow from small vessels and interstitial fluid flow in the subchondral bone and (2) subchondral ischemia with compromised gas and nutrient exchange in the articular cartilage [63]. A higher rate of blood flow is associated with an increased bone remodeling rate [63]. On the contrary, compromised blood flow in the subchondral bone could have deleterious effects on the bone and have implications for the cartilage's integrity [63]. There was a positive association between increased popliteal artery vessel wall thickness and generalized OA [62]. These evidences showed that vascular pathology plays a role in joint OA initiation and/or progression [63]. There may be common pathogenic mechanisms that affect the vascular system and joints [61]. Furthermore, most patients with knee OA who require medication for pain relief are likely to be older and at high risk for both adverse cardiovascular and gastrointestinal effects [60]. Therefore, from the point of no detrimental systemic cardiovascular effects, monochromatic IR therapy can be safely applied to elderly people with knee OA and cardiovascular diseases.

Although the present study did not support our previous hypothesis and found no evidence that monochromatic IR therapy increased knee arterial blood flow velocity in patients with knee OA, we acknowledge that there are many factors that could have affected the study results: the photosource, wavelength, power, energy density, duration of treatment, method of application (noncontact mode in contrast to contact mode), site of stimulation, size of the exposure area, and so forth. Therefore, these results cannot be considered conclusive. Our research presents a reasonable initial foray into the local and systemic cardiovascular effects of clinical monochromatic IR therapy application in patients with knee OA.

There are some limitations to the present study. First, no direct NO, prostacyclin or endothelial-derived hyperpolarizing factor productions were measured. Second, whether the increased popliteal blood flow velocity was related to the increased knee joint blood flow and/or arteriole dilatation remains uncertain.

## 5. Conclusions

 In this study, we applied monochromatic IR therapy in a double-blind, randomized, and placebo-controlled trial to subjects with knee joint OA. Our results revealed that although there was no increase in knee arterial blood flow velocity, monochromatic IR therapy produced no detrimental systemic cardiovascular effects. Therefore, it can be applied to patients with knee OA and cardiovascular diseases safely. Further studies on the effects of monochromatic IR therapy are warranted in the future using different settings for the power, wavelength, energy density, stimulation duration, and stimulation location.

## Figures and Tables

**Table 1:** Patient's basic demographics.

Variables	Groups		*P* value
	Treatment	Placebo
	*n* = 36	*n* = 35
Gender			
Female	33 (92%)	28 (80%)	0.189
Male	3 (8%)	7 (20%)	
Age (yr)	61.1 ± 9.3	61.3 ± 12.0	0.931
Body mass index (kg/m^2^)	26.4 ± 5.0	26.0 ± 4.5	0.765
Married			
Yes	28 (78%)	26 (74%)	0.730
Educational level			
Below 9th grade	21 (58%)	19 (53%)	0.650
Above 9th grade	15 (42%)	16 (47%)	
Work status			
Yes	6 (17%)	4 (12%)	0.735
Comorbidities			
Yes	18 (50%)	21 (62%)	0.322
Smoking			
Yes	0 (0%)	0 (0%)	1.000
Alcohol consumption			
Yes	3 (8%)	3 (9%)	1.000
Kellgren-Lawrence scores	2.7 ± 0.7	2.7 ± 0.7	0.962
WOMAC^∗^			
Pain	130.0 ± 87.9	116.9 ± 84.4	0.493
Stiffness	40.4 ± 47.2	40.6 ± 40.6	0.986
Physical function	413.3 ± 318.1	413.5 ± 326.8	0.999

Note: the scores are presented as the number of cases (percentage) or the mean (standard deviation) for each variable.

^∗^WOMAC: Western Ontario and McMaster University Osteoarthritis Index.

**Table 2:** Changes in heart rate, blood pressure, and blood flow velocity.

Time point	Treatment group	Placebo group	Mean between group difference	Repeated-measure ANOVA		
				*P* value (*F* value)		
	Mean (SD)	Mean (SD)	(95% confidence interval)	Group	Time	Time × group
Heart rate (beats/min)						
Initial score	69.6 (10.2)	72.2 (11.5)	−2.3 (−7.4, 2.8)	0.918	0.340	0.160
After 40 min of treatment	65.8 (8.0)	67.6 (11.2)	−1.8 (−6.4, 2.8)	(*F*_1,68_ = 0.01)	(*F*_3,204_ = 1.12)	(*F*_3,204_ = 1.74)
5 min after treatment	65.1 (8.5)	64.3 (10.4)	−0.2 (−4.8, 4.5)			
10 min after treatment	65.8 (9.2)	65.8 (10.7)	−0.9 (−5.9, 4.1)			
15 min after treatment	64.9 (10.1)	67.8 (11.7)	−3.6 (−8.7, 1.6)			
Systolic blood pressure (mmHg)						
Initial score	120.3 (16.5)	121.1 (17.6)	−0.8 (−8.9, 7.3)	0.281	0.180	0.861
After 10 min of treatment	111.8 (17.0)	114.0 (20.7)	−2.2 (−11.1, 6.8)	(*F*_1,68_ = 1.18)	(*F*_5,335_ = 1.53)	(*F*_5,335_ = 0.38)
After 20 min of treatment	109.3 (17.3)	114.6 (24.8)	−5.3 (−15.4, 4.8)			
After 30 min of treatment	111.6 (17.5)	117.3 (21.0)	−5.7 (−14.8, 3.5)			
After 40 min of treatment	112.8 (20.4)	116.4 (14.5)	−3.6 (−12.0, 4.8)			
5 min after treatment	116.1 (16.8)	117.4 (19.6)	−1.3 (−10.0, 7.3)			
15 min after treatment	114.1 (15.0)	119.9 (17.3)	−5.9 (−13.6, 1.8)			
Diastolic blood pressure (mmHg)						
Initial score	73.8 (12.0)	70.8 (11.3)	3.0 (−2.5, 8.5)	0.262	0.663	0.757
After 10 min of treatment	73.1 (12.0)	70.5 (12.0)	2.6 (−3.1, 8.3)	(*F*_1,68_ = 1.28)	(*F*_5,335_ = 0.65)	(*F*_5,335_ = 0.53)
After 20 min of treatment	69.7 (12.7)	70.1 (12.7)	−0.4 (−6.5, 5.6)			
After 30 min of treatment	70.6 (10.8)	72.9 (14.5)	−2.3 (−8.4, 3.7)			
After 40 min of treatment	72.3 (14.1)	72.8 (11.8)	−0.5 (−6.6, 5.7)			
5 min after treatment	73.7 (11.1)	74.6 (10.7)	−0.9 (−6.1, 4.3)			
15 min after treatment	73.8 (12.0)	74.9 (12.1)	−1.1 (−6.8, 4.7)			
Blood flow velocity (meters/sec)						
Initial score	36.3 (11.0)	40.5 (10.9)	−3.7 (−8.9, 1.5)	0.666	0.323	0.769
After 40 min of treatment	39.6 (10.8)	41.1 (11.3)	−1.4 (−6.6, 3.8)	(*F*_1,68_ = 0.19)	(*F*_3,204_ = 1.17)	(*F*_3,204_ = 0.38)
5 min after treatment	40.6 (11.6)	41.6 (11.5)	−1.0 (−6.4, 4.3)			
10 min after treatment	40.8 (11.6)	40.3 (10.5)	−0.2 (−5.5, 5.2)			
15 min after treatment	39.7 (10.6)	40.9 (10.5)	−1.2 (−6.2, 3.8)			

Note: scores are presented as the mean (standard deviation) for each variable.
