# Corrigendum to “Local and Systemic Cardiovascular Effects from Monochromatic Infrared Therapy in Patients with Knee Osteoarthritis: A Double-Blind, Randomized, Placebo-Controlled Study”

**DOI:** 10.1155/2017/8072517

**Published:** 2017-03-14

**Authors:** Ru-Lan Hsieh, Wei-Cheng Liao, Wen-Chung Lee

**Affiliations:** ^1^Department of Physical Medicine and Rehabilitation, Shin Kong Wu Ho-Su Memorial Hospital, 95 Wen-Chang Road, Shih-Lin District, Taipei 111, Taiwan; ^2^Department of Physical Medicine and Rehabilitation, School of Medicine, College of Medicine, Taipei Medical University, 250 Wuxing Street, Taipei 110, Taiwan; ^3^School of Nursing and Management in Gerontology, College of Nursing, Taipei Medical University, 250 Wuxing Street, Taipei 110, Taiwan; ^4^Institute of Epidemiology and Preventive Medicine, College of Public Health, National Taiwan University, 17 Xuzhou Road, Taipei 100, Taiwan

In the article titled “Local and Systemic Cardiovascular Effects from Monochromatic Infrared Therapy in Patients with Knee Osteoarthritis: A Double-Blind, Randomized, Placebo-Controlled Study” [1], affiliation number two was incomplete. The corrected affiliation is shown above.
